# Clinical Applications of Adiponectin Measurements in Type 2 Diabetes Mellitus: Screening, Diagnosis, and Marker of Diabetes Control

**DOI:** 10.1155/2018/5187940

**Published:** 2018-07-05

**Authors:** Nabila A. Abdella, Olusegun A. Mojiminiyi

**Affiliations:** ^1^Department of Medicine, Faculty of Medicine, Kuwait University, Safat, Kuwait; ^2^Department of Pathology, Faculty of Medicine, Kuwait University, Safat, Kuwait

## Abstract

**Background:**

Adipose tissue-derived adiponectin has pleiotropic protective effects with suppression of inflammatory and metabolic derangements that may result in insulin resistance, metabolic syndrome, type 2 diabetes mellitus (T2DM), and cardiovascular disease. The aim of this study was to evaluate adiponectin as a diagnostic marker of T2DM and diabetes control.

**Methods:**

Fasting adiponectin, insulin, glucose, and HbA1c were determined in 376 patients with known T2DM and 575 subjects with undiagnosed diabetes but with family history of T2DM. Clinical and anthropometric data were recorded. Subjects were classified on the basis of degree of adiposity, insulin resistance (IR), and achievement of target HbA1c levels. Receiver operating characteristic (ROC) curve analysis was used to examine the diagnostic performance for undiagnosed DM.

**Results:**

In undiagnosed subjects, adiponectin was significantly lower in subjects with IR and diabetic subjects compared with those without. The area under the adiponectin ROC curve for diagnosis of DM was 0.740. In known T2DM subjects, those with good control had significantly higher adiponectin (8.6 versus 7.4 *μ*g/mL) compared to subjects with poor control.

**Conclusions:**

Adiponectin levels are associated with better glycemic control and could be a useful adjunct for screening for IR and T2DM. Therapeutic measures that increase adiponectin levels might be valuable targets for improving diabetes control and decreasing complications.

## 1. Introduction

Type 2 diabetes mellitus (T2DM) is highly prevalent and is one of the leading causes of mortality and morbidity worldwide. T2DM is characterized by insulin resistance or impaired insulin secretion often in association with obesity which causes insulin resistance through secretion of various adipocyte-derived proteins. Adiponectin, a bioactive adipocytokine exclusively secreted by mature adipocytes in adipose tissue possesses anti-inflammatory, antiatherogenic, and insulin-sensitizing properties. It is the most abundant adipocytokine synthesized by adipocytes and the only adipose-specific protein that is negatively regulated in obesity [1–3]. Various cross-sectional studies have documented the association of low adiponectin levels with obesity, insulin resistance, metabolic syndrome (MetS), and progression from prediabetes to T2DM.

The pleiotropic protective effects of the adiponectin occurs via several postulated mechanisms that could, potentially, reduce the risk of T2DM and its associated complications. Some of the multiple anti-inflammatory and antiatherogenic effects of adiponectin involve vascular endothelial cell survival and activation through inhibition of TNF-*α* signaling [4] and regulation of endothelial nitric oxide synthase (eNOS). Adiponectin exerts potent insulin-sensitizing action through fatty acid oxidation, increased energy consumption, and stimulation of insulin secretion [2, 5]. There is strong accumulating evidence from several prospective studies that showed low adiponectin levels as a predictor of the incidence of T2DM [6, 7]. Adiponectin has been shown to significantly correlate inversely with obesity, hypertension, dyslipidemia, fasting plasma glucose levels, and insulin resistance [8–11], which are known risk factors for subsequent development of T2DM. Given the prominent biological functions and associations of adiponectin as a protective adipocytokine against T2DM, this study assesses the potential roles of adiponectin as a useful clinical diagnostic indicator of incident diabetes among individuals at increased risk as well as diabetes control among T2DM patients.

## 2. Materials and Methods

### 2.1. Subjects and Clinical Features

The study subjects were recruited from polyclinics, specialized diabetes clinics, and hospitals in Kuwait where subjects with T2DM were advised to invite their first-degree relatives (FDR) for screening to assess their risks of developing T2DM as described previously [12]. 575 apparently healthy FDR of patients with T2DM were studied. To evaluate adiponectin as a marker of DM control, we studied 376 patients with known T2DM duration of 12.4 ± 8.1 years. Prediabetes and T2DM were confirmed according to the criteria of the American Diabetes Association [13]. For the first-degree relatives, criteria for inclusion in the study were both parents, one parent, and/or a sibling with T2DM and absence of any hematologic, genetic (especially presence of hemoglobin variants), and illness-related factors that could affect or interfere with the estimation of HbA1c. None of the study subjects was taking any medication at the time of the study. None of the study subjects had a history of surgery on reproductive organs (e.g., oophorectomy), and female subjects were specifically asked if their menstruation had stopped. None of the female study subjects was menopausal at the time of the study. The study was approved by the Ethics Committees of the Faculty of Medicine, Kuwait University, and the Ministry of Health, Kuwait, and performed in accordance with the Declaration of Helsinki. All subjects gave informed voluntary consent to participate in the study.

Height was measured to the nearest 0.1 cm and body weight to the nearest 0.1 kg using a stadiometer, and the body mass index (BMI) (kg/m^2^) was calculated [12]. The body mass index (BMI) was calculated according to the following formula: weight in kilograms divided by the square of the height in meters. Patients with BMI > 30 kg/m^2^ are classified as obese and those with BMI < 25 are classified as normal. Those with BMI > 24.9 to <30 are classified as overweight. Waist circumference (WC) was measured half way between the xiphisternum and the umbilicus at the point corresponding to the maximal abdominal protuberance. Two consecutive measurements of systolic blood pressure (SBP) and diastolic blood pressure (DBP) were taken from each subject after at least 10 min rest.

### 2.2. Laboratory Methods

#### 2.2.1. Assays

As described previously [12], fasting plasma adiponectin was measured using a commercially available enzyme-linked immunoassay (ELISA) kit (Linco Research, Missouri, USA) with a sensitivity of 0.39 *μ*g/mL. The intra- and interassay coefficients of variation on pooled plasma specimen with adiponectin concentration of 8.2 *μ*g/mL were 4.7% and 6.8%, respectively.

Fasting serum insulin was determined on an automated analyzer Beckman DXI 800 (Beckman Corporation) using the paramagnetic particle chemiluminescence immunoassay method [12]. Insulin resistance was calculated using the homeostasis model assessment (HOMA-IR) using the HOMA2 calculator (version 2.2.2) downloaded from https://www.dtu.ox.ac.uk/homacalculator/download.php (Diabetes Trials Unit, Oxford). HOMA-IR > 2 was used as the cut-off point for determination of insulin resistance [14]. The HOMA2 calculator also gives estimates of steady-state beta cell function (%B) and insulin sensitivity (%S).

HbA1c levels were measured using high-performance liquid chromatography on a TOSOH G8 analyzer (Tosoh Corporation, Tokyo, Japan) [12]. HbA1c values or fasting plasma glucose (FPG) based on ADA diagnostic criteria [13] was used to categorize the subjects as follows: normal subjects—HbA1c < 5.7% (<39 mmol/mol) or FPG < 5.6 mmol/L; subjects with prediabetes—HbA1c 5.7%–6.4% (39–46 mmol/mol) or FPG 5.6–6.9 mmol/L; subjects with diabetes—HbA1c ≥ 6.5% (≥48 mmol/mol) or FPG ≥ 7.0 mmol/L.

FPG, alanine aminotransferase (ALT), total cholesterol (TC), triglycerides (TG), high-density lipoprotein cholesterol (HDL-C), apolipoprotein A1 (Apo-A1), and apolipoprotein B (Apo B) were analyzed on an automated analyzer (Beckman DXC 800, Beckman Corporation) [12]. Low-density lipoprotein cholesterol (LDL-C) was calculated using the Friedewald formula [15]. The formula is valid as long as TG ≤ 4.5 mmol/L.

### 2.3. Statistical Methods

IBM SPSS 19.0 software (IBM) was used for statistical analysis. Data are presented as mean ± standard deviation (SD) unless otherwise specified. The Kolmogorov–Smirnov goodness-of-fit test was to test for normality of the data. Several variables (insulin, HOMA-IR, B%, and TG) that diverged significantly from normal distribution were log transformed when parametric tests were used. Comparisons between two groups were performed with the Mann–Whitney *U* test, and the Kruskal-Wallis analysis of variance was used to compare between more than two groups. The chi-square test was used to compare categorical variables. To determine the predictors of adiponectin, we performed linear and multivariate regression analysis with adiponectin as the dependent variable. All cardiometabolic variables were included in the multivariate stepwise regression model. Binary logistic regression analysis was performed to estimate the odds ratios (ORs) and 95% confidence intervals (CIs) for the association between adiponectin and T2DM. We performed receiver operating characteristic (ROC) analysis on the usefulness of adiponectin for the detection of diabetes. *p* < 0.05 was considered statistically significant for all analyses.

## 3. Results

### 3.1. General Results

**Figure 1:**
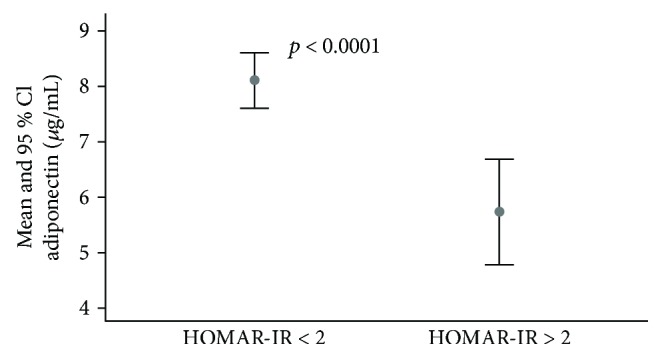
Adiponectin levels in subjects with undiagnosed diabetes grouped by homeostasis model assessment of insulin resistance. CI = confidence interval.

73 of 575 study subjects were found to have previously undiagnosed T2DM. Tables 1 and 2 summarize clinical and anthropometric parameters of study subjects screened for glycemic status grouped by degree of adiposity and T2DM, respectively. Subjects who were obese had significantly higher blood pressure, lipid profile, ALT, insulin, B%, HOMA-IR, fasting glucose, and HbA1c levels and lower S% compared to normal individuals. Adiponectin levels were significantly lower with increasing degree of adiposity (Table 1). In Figure 1, FDR screened for undiagnosed diabetes were categorized according to their state of IR. Subjects who were insulin resistant were found to have significantly reduced adiponectin concentrations (5.7 versus 8.1 *μ*g/mL) compared to subjects without IR. In Table 2, FDR diagnosed with T2DM had significantly higher WC, SBP, ALT, insulin, B%, and HOMA-IR but significantly lower S% and adiponectin levels (6.9 versus 8.6 *μ*g/mL) compared to those subjects who did not develop T2DM.

Table 3 shows the clinical, anthropometric, and metabolic characteristics of known T2DM patients (*n* = 376) grouped according to their achievement of target HbA1c levels. T2DM patients with poor glycemic control (>53 mmol/mol) had higher ALT, B%, and HOMA-IR but significantly lower adiponectin levels (7.4 versus 8.6 *μ*g/mL) compared to subjects with good control (<53 mmol/mol).

### 3.2. Predictors of Circulating Adiponectin

The associations of adiponectin with cardiometabolic variables are shown in Table 4. With the exception of HbA1c, adiponectin was significantly associated with cardiometabolic variables. However, when all the cardiometabolic variables were included in the full model of regression analysis, the strongest significant predictors of circulating adiponectin were waist circumference, HDL-cholesterol, apolipoprotein A1, apolipoprotein B, and HOMA-IR.

### 3.3. Regression Analysis

Binary logistic regression analysis showed that adiponectin was significantly associated with T2DM with an odds ratio of 0.88 [95% confidence interval (CI) 0.80–0.96; *p* = 0.007].

#### 3.3.1. Performance Characteristics for Detection of T2DM

**Figure 2:**
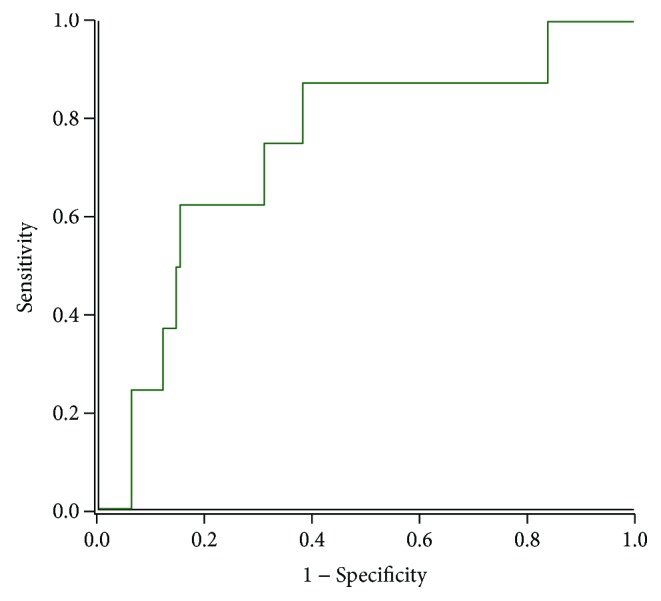
Receiver operating characteristic analysis of the usefulness of adiponectin for the diagnosis of type 2 diabetes mellitus.

Using the ADA glucose and HbA1c diagnostic criteria as reference, ROC curve (Figure 2) analyses for the use of adiponectin to detect T2DM showed that the area under the adiponectin curve was 0.740 (95% CI 0.570–0.910). At the cut-off point of 7.5 *μ*g/mL, the diagnostic sensitivity and specificity of adiponectin for T2DM were 88% and 51%, respectively.

## 4. Discussion

This study demonstrates the potential clinical significance of adiponectin measurements in T2DM. Our results confirm and extend those of several studies that demonstrated the protective role of high adiponectin levels with lower risk of T2DM and association of low adiponectin levels with risk factors for T2DM [16, 17] and subsequent cardiovascular complications associated with the disease [18, 19].

The significant progressive decrease in adiponectin (Table 1) in line with increasing degree of insulin resistance (Table 1 and Figure 1) with increasing degrees of obesity indicate the utility of adiponectin as a screening tool. Obesity is an important determinant of insulin resistance and a known risk factor for development of T2DM and cardiovascular disease. The significant associations of adiponectin with cardiometabolic risk factors (Table 4) show the usefulness for the identification of high-risk first-degree relatives of T2DM patients who tend to exhibit a higher propensity to be insulin resistant [20]. Hypoadiponectinemia has been shown to precede a decrease in insulin sensitivity [5] as well as predict progression from normoglycemia to prediabetes [21]. These studies and our findings highlight the potential of adiponectin as a screening tool that could be used to monitor progression from prediabetes to diabetes and associated complications.

The usefulness of adiponectin for the detection of the metabolic syndrome had been shown in a previous study [14]. The present study on subjects at high risk of T2DM has shown that adiponectin has good performance characteristics for the detection of previously undiagnosed T2DM. The diagnostic sensitivity, specificity and area under the ROC curve (Figure 2) show that adiponectin could be a useful adjunct for the diagnosis of T2DM in a population screen for T2DM. Even after adjustment for confounding risk factors, the binary logistic regression association of adiponectin with T2DM with an odds ratio of 0.88 further highlights low adiponectin as a strong predictor of incident T2DM [19].

One of the interesting findings in our study is the significant difference in adiponectin concentrations between T2DM subjects with good glycemic control and poor glycemic control (Table 3). The association of higher adiponectin levels with better glycemic control suggests that therapeutic modalities that increase adiponectin levels may be valuable targets for management of T2DM. Methods that enhance or mimic adiponectin levels have been shown to be effective therapeutic strategies for improving diabetes control, treatment of insulin resistance, and other metabolic abnormalities associated with T2DM. Thiazolidinediones are a class of antidiabetic drugs, known to exert an insulin-sensitizing effect, mediated partly by upregulating plasma adiponectin levels [2]. Several other drugs like statins, angiotensin-converting enzyme inhibitors, and angiotensin II receptor blockers that target adiponectin synthesis have also been reported to improve glucose tolerance and ameliorate insulin resistance [22]. However, although adiponectin is present at relatively high amounts in circulation and can be easily measured, the efficacy of adiponectin as a potentially useful therapeutic agent needs to be demonstrated experimentally and in clinical trials.

One of the main limitations of this study is that it is a cross-sectional study which does not help establish a causal relation between low adiponectin and T2DM development. Additionally, study participants used in our study were those at high risk for developing T2DM; thus, the associations observed with adiponectin and T2DM risk may not be applicable to those without family history of T2DM. Our study does not provide data on adiponectin multimeric complexes particularly high molecular weight (HMW) adiponectin, which has been shown to be the most biologically active form. However, others have reported similar associations of both total adiponectin and HMW with incident T2DM [23] and cardiometabolic risk factors [24].

## 5. Conclusion

Adiponectin levels are associated with incident diabetes and glycemic control and could be useful adjuncts for screening for IR and T2DM. The significant associations of adiponectin levels with clinical and cardiometabolic parameters reveal its potential as a biomarker in assessment of prediabetic state and T2DM screening.

## Figures and Tables

**Table 1:** Clinical and anthropometric characteristics of study subjects screened for glycemic status grouped by degree of adiposity.

BMI (kg/m^2^)	Normal (BMI < 25)	Overweight (24.9–30)	Obese (>30)	*p* (Kruskal-Wallis)
Age (yrs)	25.7 ± 8.2	28.9 ± 8.9	31.8 ± 8.8	<0.0001
WC (cms)	83.8 ± 9.2	95 ± 9	108.7 ± 12.6	<0.0001
SBP (mm/Hg)	109 ± 10	115 ± 15	116 ± 13	0.001
DBP (mm/Hg)	69 ± 8	72 ± 8	74 ± 7	<0.0001
TC (mmol/L)	4.48 ± 0.82	4.93 ± 0.98	5.15 ± 1.13	<0.0001
TG (mmol/L)	0.81 ± 0.54	1.09 ± 0.72	1.32 ± 0.95	<0.0001
HDL-C (mmol/L)	1.25 ± 0.36	1.18 ± 0.33	1.16 ± 0.31	0.23
LDL-C (mmol/L)	2.79 ± 0.71	3.04 ± 0.83	3.34 ± 0.92	<0.0001
ALT (IU/L)	22.52 ± 17.05	24.63 ± 15.34	27.37 ± 16.57	<0.0001
Insulin (*μ*U/mL)	5.38 ± 4.68	6.8 ± 6.07	10.86 ± 9.47	<0.0001
S%	143.19 ± 59.44	122.24 ± 54.92	94.4 ± 51.81	<0.0001
B%	87.55 ± 36.83	100.43 ± 67.71	111.05 ± 54.5	<0.0001
HOMA-IR	0.9 ± 0.65	1.07 ± 0.74	1.46 ± 1.04	<0.0001
Glucose (mmol/L)	5 ± 0.99	5.05 ± 0.66	5.22 ± 1.03	0.03
HbA1c (%)	5.88 ± 0.83	5.87 ± 0.62	8.75 ± 26.39	0.001
Adiponectin (*μ*g/mL)	8.95 ± 3.82	8.57 ± 5.12	6.82 ± 2.97	<0.0001

Abbreviations: WC: waist circumference; SBP: systolic blood pressure; DBP: diastolic blood pressure; TC: total cholesterol; TG: triglyceride; HDL-C: high-density lipoprotein; LDL-C: low-density lipoprotein; ALT: alanine aminotransferase; S%: insulin sensitivity; B%: beta cell function; HOMA-IR: homeostasis model assessment of insulin resistance; HbA1c: hemoglobin A1c. Data are presented as mean ± standard deviation

**Table 2:** Clinical and anthropometric characteristics of subjects screened for type 2 diabetes mellitus.

HbA1c	Normal (<5.7%)	Undiagnosed diabetes (≥6.5%)	*p* (Mann–Whitney *U*)
Age (yrs)	28.2 ± 8.6	32 ± 9.7	0.019
WC (cms)	93.6 ± 16.0	104.6 ± 14.7	<0.0001
SBP (mm/Hg)	113 ± 13	120 ± 17	0.005
DBP (mm/Hg)	73 ± 9	73 ± 10	0.37
TC (mmol/L)	4.65 ± 0.91	5.02 ± 0.9	0.001
TG (mmol/L)	1.0 ± 0.71	1.28 ± 0.69	<0.0001
HDL-C (mmol/L)	1.20 ± 0.34	1.07 ± 0.26	0.007
LDL-C (mmol/L)	2.96 ± 0.76	3.32 ± 0.81	<0.0001
ALT (IU/L)	23.42 ± 16.57	30.91 ± 19.24	<0.0001
Insulin (*μ*U/mL)	7.46 ± 6.95	11.87 ± 11.33	<0.0001
S%	119.31 ± 56.71	91.43 ± 54.42	0.002
B%	101.52 ± 54.32	110.15 ± 70.08	0.1
HOMA-IR	1.12 ± 0.79	1.66 ± 1.42	0.002
Glucose (mmol/L)	4.97 ± 0.64	5.91 ± 1.76	<0.0001
HbA1c (%)	5.63 ± 0.40	7.58 ± 1.14	<0.0001
Adiponectin (*μ*g/mL)	8.57 ± 4.42	6.92 ± 2.86	<0.0001

Abbreviations: WC: waist circumference; SBP: systolic blood pressure; DBP: diastolic blood pressure; TC: total cholesterol; TG: triglyceride; HDL-C: high-density lipoprotein; LDL-C: low-density lipoprotein; ALT: alanine aminotransferase; S%: insulin sensitivity; B%: beta cell function; HOMA-IR: homeostasis model assessment of insulin resistance; HbA1c: hemoglobin A1c. Data are presented as mean ± standard deviation.

**Table 3:** Clinical and anthropometric characteristics of type 2 diabetic subjects grouped by achievement of target HbA1c.

HbA1c	<53 mmol/mol	>53 mmol/mol	*p* Mann–Whitney *U*
Age	55.4 ± 13.5	57.8 ± 9.4	NS
WC (cms)	102.6 ± 13.2	107 ± 12.9	NS
BMI (kg/m^2^)	30.3 ± 7.7	32.8 ± 6.0	0.05
SBP (mm/Hg)	132 ± 28	132 ± 20	NS
DBP (mm/Hg)	82 ± 13	82 ± 12	NS
TC (mmol/L)	4.88 ± 1.11	5.02 ± 1.18	NS
TG (mmol/L)	1.54 ± 1.06	1.75 ± 1.19	NS
HDL-C (mmol/L)	1.21 ± 0.34	1.11 ± 0.29	0.04
LDL-C (mmol/L)	3.07 ± 0.95	3.04 ± 0.98	NS
ALT (IU/L)	21.91 ± 12.72	34.15 ± 177.75	0.02
Insulin (*μ*U/mL)	11.51 ± 14.74	17.15 ± 25.48	NS
S%	88.05 ± 41.30	73.46 ± 50.21	0.03
B%	77.08 ± 53.19	43.40 ± 37.69	<0.0001
HOMA-IR	1.51 ± 1.04	2.31 ± 2.0	0.03
Glucose (mmol/L)	6.73 ± 2.03	11.23 ± 4.08	<0.0001
HbA1c (%)	6.34 ± 0.47	10.69 ± 2.53	<0.0001
Adiponectin (*μ*g/mL)	8.58 ± 4.5	7.37 ± 4.35	0.04

Abbreviations: WC: waist circumference; BMI: body mass index; SBP: systolic blood pressure; DBP: diastolic blood pressure; TC: total cholesterol; TG: triglyceride; HDL-C: high-density lipoprotein; LDL-C: low-density lipoprotein; ALT: alanine aminotransferase; S%: insulin sensitivity; B%: beta cell function; HOMA-IR: homeostasis model assessment of insulin resistance; HbA1c: hemoglobin A1c; NS: not significant (*p* > 0.05). Data are presented as mean ± standard deviation

**Table 4:** Multivariate regression analysis of the associations of cardiometabolic variables with adiponectin concentration as a dependent variable in subjects screened for type 2 diabetes mellitus.

	*β* coefficient	95% confidence interval	*p*	*β* ^∗^ coefficient	95% confidence interval	*p*
WC (cms)	−0.341	−0.114 to −0.066	<0.0001	−0.314	−0.178 to −0.008	0.023
BMI (kg/m^2^)	−0.224	−0.211 to −0.062	<0.0001	0.220	−.028 to 0.348	0.059
SBP (mm/Hg)	−0.151	−0.086 to −0.018	0.003	−0.045	−0.082 to 0.055	0.695
DBP (mm/Hg)	−0.160	−0.138 to −0.033	0.002	−.097	−0.178 to 0.074	0.415
TC (mmol/L)	−0.187	−1.325 to −0.421	<0.0001	−0.145	−2.701 to 1.343	0.507
TG (mmol/L)	−0.291	−2.269 to −1.159	< 0.0001	−0.135	−1.927 to 0.346	0.172
HDL-C (mmol/L)	0.338	3.170 to 5.578	<0.0001	0.309	1.397 to 6.588	0.003
LDL-C (mmol/L)	−0.240	−1.809 to −0.759	<0.0001	−0.161	−2.865 to 1.085	0.376
ApoA1 (g/L)	0.136	0.169 to 3.673	0.032	−.462	−13.608 to −2.371	0.006
Apo B (g/L)	−0.269	−4.647 to −1.995	< 0.0001	−0.271	−1.165 to −0.502	<0.0001
HOMA-IR	−0.252	−1.550 to −0.548	<0.0001	−0.166	−1.377 to −0.012	004
Glucose (mmol/L)	−0.173	−1.247 to −0.352	<0.0001	−0.097	−0.791 to 0.039	0.076
HbA1c (mmol/mol)	−0.061	−0.050 to 0.012	0.227	0.055	−0.672 to 1.286	0.054

Abbreviations: WC: waist circumference; BMI: body mass index; TC: total cholesterol; TG: triglyceride; HDL-C: high-density lipoprotein; LDL-C: low-density lipoprotein; Apo A1: apolipoprotein A1; Apo B: apolipoprotein B; HbA1c: hemoglobin A1c; SBP: systolic blood pressure; DBP: diastolic blood pressure; HOMA-IR: homeostasis model assessment of insulin resistance; *β*^∗^: inclusion of all cardiometabolic variables in the regression model.
